# Comparison of Medical Cannabis Use Reported on a Confidential Survey vs Documented in the Electronic Health Record Among Primary Care Patients

**DOI:** 10.1001/jamanetworkopen.2022.11677

**Published:** 2022-05-23

**Authors:** Gwen T. Lapham, Theresa E. Matson, David S. Carrell, Jennifer F. Bobb, Casey Luce, Malia M. Oliver, Udi E. Ghitza, Clarissa Hsu, Kendall C. Browne, Ingrid A. Binswanger, Cynthia I. Campbell, Andrew J. Saxon, Ryan Vandrey, Gillian L. Schauer, Rosalie Liccardo Pacula, Michael A. Horberg, Steffani R. Bailey, Erin A. McClure, Katharine A. Bradley

**Affiliations:** 1 Kaiser Permanente Washington Health Research Institute, Seattle; 2 Department of Health Systems and Population Health, University of Washington, Seattle; 3 Center for the Clinical Trials Network, National Institute on Drug Abuse, National Institutes of Health, Bethesda, Maryland; 4 Center of Excellence in Substance Addiction Treatment and Education, Veteran Affairs Puget Sound Health Care System, Seattle, Washington; 5 Kaiser Permanente Colorado Institute for Health Research, Denver; 6 Colorado Permanente Medical Group, Denver; 7 Kaiser Permanente Northern California Division of Research, Oakland; 8 Johns Hopkins University School of Medicine, Baltimore, Maryland; 9 Addictions, Drug & Alcohol Institute, University of Washington, Seattle; 10 Price School of Public Policy, University of Southern California, Los Angeles; 11 Leonard D Schaeffer Center for Health Policy & Economics, University of Southern California, Los Angeles; 12 Kaiser Permanente Mid-Atlantic Permanente Research Institute, North Bethesda, Maryland; 13 Department of Family Medicine, Oregon Health & Science University, Portland; 14 Medical University of South Carolina College of Medicine, Charleston; 15 Department of Medicine, University of Washington, Seattle

## Abstract

**Importance:**

Patients who use cannabis for medical reasons may benefit from discussions with clinicians about health risks of cannabis and evidence-based treatment alternatives. However, little is known about the prevalence of medical cannabis use in primary care and how often it is documented in patient electronic health records (EHR).

**Objective:**

To estimate the primary care prevalence of medical cannabis use according to confidential patient survey and to compare the prevalence of medical cannabis use documented in the EHR with patient report.

**Design, Setting, and Participants:**

This study is a cross-sectional survey performed in a large health system that conducts routine cannabis screening in Washington state where medical and nonmedical cannabis use are legal. Among 108 950 patients who completed routine cannabis screening (between March 28, 2019, and September 12, 2019), 5000 were randomly selected for a confidential survey about cannabis use, using stratified random sampling for frequency of past-year use and patient race and ethnicity. Data were analyzed from November 2020 to December 2021.

**Exposures:**

Survey measures of patient-reported past-year cannabis use, medical cannabis use (ie, explicit medical use), and any health reason(s) for use (ie, implicit medical use).

**Main Outcomes and Measures:**

Survey data were linked to EHR data in the year before screening. EHR measures included documentation of explicit and/or implicit medical cannabis use. Analyses estimated the primary care prevalence of cannabis use and compared EHR-documented with patient-reported medical cannabis use, accounting for stratified sampling and nonresponse.

**Results:**

Overall, 1688 patients responded to the survey (34% response rate; mean [SD] age, 50.7 [17.5] years; 861 female [56%], 1184 White [74%], 1514 non-Hispanic [97%], and 1059 commercially insured [65%]). The primary care prevalence of any past-year patient-reported cannabis use on the survey was 38.8% (95% CI, 31.9%-46.1%), whereas the prevalence of explicit and implicit medical use were 26.5% (95% CI, 21.6%-31.3%) and 35.1% (95% CI, 29.3%-40.8%), respectively. The prevalence of EHR-documented medical cannabis use was 4.8% (95% CI, 3.45%-6.2%). Compared with patient-reported explicit medical use, the sensitivity and specificity of EHR-documented medical cannabis use were 10.0% (95% CI, 4.4%-15.6%) and 97.1% (95% CI, 94.4%-99.8%), respectively.

**Conclusions and Relevance:**

These findings suggest that medical cannabis use is common among primary care patients in a state with legal use, and most use is not documented in the EHR. Patient report of health reasons for cannabis use identifies more medical use compared with explicit questions about medical use.

## Key Points

**Question:**

What is the prevalence of patient-reported explicit (ie, medical use) and implicit (ie, health reasons for use) medical cannabis use, and how does electronic health record documentation compare with patient report of medical use?

**Findings:**

In this survey study, among 1688 primary care patients, 26.5% reported explicit and 35.1% reported implicit medical use of cannabis. The prevalence of medical use documented in the electronic health record was 4.8%, missing most medical cannabis use reported by patients.

**Meaning:**

These findings suggest that asking about use of cannabis for managing pain, sleep, mood, or other health concerns may increase recognition and documentation of medical cannabis use.

## Teaser

This survey study compares the use of cannabis for medical purposes as reported in electronic health records (EHRs) with use reported in a confidential survey.

## Introduction

Cannabis and cannabinoid use in the US is prevalent and increasing.^1,2^ A majority of states have legalized medical cannabis use, and among these, 18 have legalized nonmedical use.^3,4^ A recent study found the prevalence of past-year cannabis use among primary care patients routinely screened for cannabis use in a state with legal nonmedical use was greater than 20%.^5^

Documentation of patients’ medical cannabis use in the electronic health record (EHR) can support patient-clinician discussions of the risks of cannabis use and exploration of treatment alternatives. Patients use cannabis for a variety of health conditions,^6,7,8,9,10^ and although evidence suggests potential benefit for neuropathic pain, appetite, nausea and vomiting, spasticity, and short-term sleep outcomes, most health conditions for which patients use cannabis have insufficient or nonexistent evidence of benefit, potential contraindications, and more effective first-line treatment options.^11,12,13^ Moreover, cannabis use has known risks, including increased risk of cannabis and other substance use disorders, mental health disorders, acute care utilization, and withdrawal.^13,14,15,16,17,18,19,20,21^

The prevalence of EHR-documented medical cannabis use may be low in comparison to self-reported prevalence.^22,23^ The recent study of patients routinely screened for past-year cannabis use^5^ also found that only 2% of patients had documentation of medical cannabis use in their EHR over a 1-year period, including documentation of explicit (ie, medical use) and implicit (ie, use to self-manage a health condition or symptom) medical use.

 To understand how EHR documentation of medical use compares with patient report, we used a confidential patient survey to (1) estimate the prevalence of explicit and implicit medical cannabis use among primary care patients in a state with legal nonmedical cannabis use, and (2) compare the performance of EHR-documented medical cannabis use with patient-reported medical cannabis use on the survey as the reference standard.

## Methods

### Study Sample

This survey study received approval and waivers of consent (to identify eligible sample), documentation of informed consent (for survey respondents), and HIPAA (Health Insurance Portability and Accountability Act) authorization from the Kaiser Permanente Washington (KPWA) Health Research Institute institutional review board. This study follows the American Association for Public Opinion Research (AAPOR) reporting guideline for mixed-mode surveys.^24^

The eligible primary care sample included adult (aged ≥18 years) patients who completed a single-item cannabis screen (eg, index cannabis screen) between January 28, 2019, and September 12, 2019, during routine primary care in KPWA (Figure).^25,26^ KPWA is a large integrated health insurance plan and delivery system in Washington State, where nonmedical cannabis use has been legal since 2012 and can be purchased for medical use without physician authorization.^27^ Patients who were KPWA employees (approximately 4%), needed an interpreter (2.6%), lived outside Washington State (<1%), were recently deceased (<1%), or opted out of EHR research (<1%) were excluded. The screen, adapted from a validated alcohol screen,^28,29^ assessed the frequency of past-year cannabis use with the question, “How often in the past year have you used marijuana?” with response options of never, less than monthly, monthly, weekly, or daily/almost daily. More than 80% of KPWA primary care patients are screened annually for marijuana use, without reference to medical or nonmedical use.^26,30^

**Figure.**
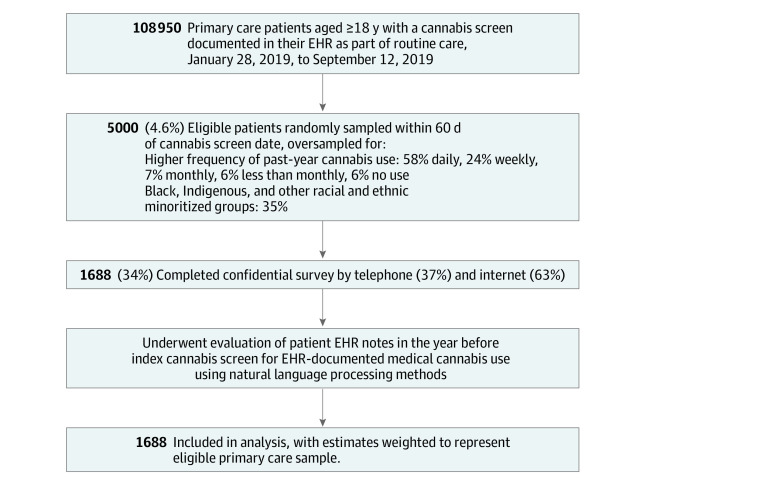
Flow Diagram of Study Sample Patients who were Kaiser Permanente Washington employees, needed an interpreter, lived outside Washington State, were recently deceased, or opted out of EHR research were excluded. Other racial and ethnic minoritized groups includes American Indian, Alaskan Native, Asian, Pacific Islander, Black/African American, Hispanic/Latinx, and Multiracial. EHR indicates electronic health record.

Among 108 950 primary care patients eligible from March 28, 2019, to September 12, 2019, 5000 (4.6% of eligible), including patients who reported no past-year cannabis use, were randomly sampled within 60 days of their index cannabis screen date to ensure proximity of survey to index screen (Figure). Patients were oversampled for higher frequency of past-year cannabis use and for members of racial and ethnic minoritized groups, including Hispanic/Latinx, to ensure adequate representation of racial and ethnic minoritized groups and those who use cannabis. Sampled patients received a mailed invitation, information about confidentiality, request to access patient EHR data, web-survey URL, unique study identifier, a $2 incentive, and notification of receipt of compensation ($20) for survey completion. Follow-up reminder calls offered telephone completion, prompts to complete online, and/or an offer to email the survey link. Patients acknowledged informed consent before completing the survey.

### Data Collection

Data were obtained from patient’s survey responses and EHRs. The survey, developed with an expert panel of cannabis researchers (G.L.S., R.L.P., R.V., E.A.M., S.R.B., and M.A.H.) and co-investigators (U.E.G., C.H., K.C.B., I.A.B., C.I.C., A.J.S., K.A.B., and G.T.L.), was designed to assess primary care patient cannabis use, including medical use. Question selection was iterative to achieve consensus and included previously published medical use questions,^22^ as well as new items, including a question to assess the patient’s health reasons for use (implicit medical cannabis use). The survey was pilot tested for feasibility and acceptability with a convenience sample, including coauthors and KPWA research staff and acquaintances, before administration. The survey included 75 items (eAppendix in the Supplement) and took an average of 20 minutes. Respondents were instructed to consider a comprehensive definition of cannabis/cannabinoid use, including marijuana, cannabis concentrates, edibles, lotions, ointments, and tinctures made with cannabis, as well as CBD-only (cannabidiol) products.

Demographic data (eg, age, sex, race, ethnicity, and insurance), collected from patients by KPWA and documented in the EHR before or at the time of the index cannabis screen, were obtained for both survey respondents and nonrespondents to allow for nonresponse weighting. EHR data on free-text documentation of medical cannabis use and *International Statistical Classification of Diseases and Related Health Problems, Tenth Revision (ICD-10)* codes for diagnoses in the year before the index screen date were also obtained for respondents.

### Measures

#### Patient Report of Medical Cannabis Use on a Confidential Survey

The survey first asked 2 questions about the frequency and recency of past-year cannabis use (the only 2 required for survey completion). Those with a response other than never for past-year use (ie, indicating patient report of past-year cannabis use) were asked additional questions, including 2 about past-year medical use. The primary survey measure of interest, which was used previously,^22,31^ asked explicitly about medical and nonmedical cannabis use (without defining medical use), as follows: “When you used marijuana/cannabis during the past year, was it: (1) for medical reasons, (2) for nonmedical reasons, or (3) both medical and nonmedical reasons?” Patients were considered to have patient report of explicit medical cannabis use if they reported any medical use (ie, both medical and nonmedical or only medical). Another question, developed as a measure of implicit medical or health reasons for cannabis use, asked patients about their reasons for use with the question, “During the past year, have you used marijuana/cannabis to help you manage any of the following? [Check all that apply]”. Response options, based on health reasons patients frequently report for using cannabis,^6,7,8,9,10^ included separate yes/no checkboxes for pain, muscle spasm, seizures, nausea or vomiting, sleep, stress, appetite, worry or anxiety, depression or sadness, focus or concentration, other symptoms (write-in option), and none*.* A binary indicator of patient report of implicit medical use was created for patient report of any of the 11 reasons, including other symptoms.

#### EHR-Documented Medical Cannabis Use

Patients were categorized as having EHR-documented medical cannabis use if more than 1 EHR note and/or an *ICD-10* diagnosis indicated medical cannabis use. To identify medical cannabis use in respondent EHRs, a binary indicator of past-year medical cannabis use was created from EHR text records using methods described previously.^5,32^ Medical use was defined by clinician recommendation or characterized by clinician or patient as use to manage a health condition or symptom, explicitly (eg, medical marijuana most days) or implicitly (eg, cannabis for low back pain). Although the EHR did not prompt clinicians to document medical use, clinicians could document reasons for use in notes. In brief, all patient EHR notes, within the year before the index cannabis screen date, were evaluated using an automated machine-learned natural language processing (NLP) algorithm applied to EHR notes to (1) identify cannabis and cannabinoid terms (eg, marijuana, cannabis, THC [tetrahydrocannabinol], CBD, or pot); (2) flag nonrelevant cannabis mentions (eg, negated, historical, and hypothetical); and (3) identify relevant mentions of implicit and/or explicit medical cannabis use, according to previously defined terms.^32^ The automated NLP algorithm, validated specifically for medical cannabis use, achieved high specificity (94%) and limited sensitivity (67%) in a validation study^32^ and was augmented with NLP-assisted manual review to identify medical cannabis use mentions not captured by the algorithm.

#### Other Measures

Other survey measures included patient report of frequency of past-year cannabis use, marital status, and type of residence. Other EHR measures included EHR-documented frequency of past-year use (from index cannabis screen) and number of days with any free-text documentation (ie, note-days), log-transformed, to account for greater opportunity for EHR-documentation associated with greater health care use.

### Statistical Analysis

All analyses were weighted to account for stratified random survey sampling and nonresponse, unless noted otherwise, so that prevalence estimates were representative of the eligible primary care sample (Figure). To account for survey sampling, weights were created for the inverse proportion of eligible patients randomly sampled within each of 10 strata resulting from 5 cannabis screen responses and the indicator for patients from racial and ethnic minoritized groups (eTable 1 in the Supplement).^33,34^ Inverse probability weights were estimated using logistic regression to account for differences between respondents and nonrespondents according to demographic characteristics available at sampling. The 2 weights, multiplied, were applied to survey respondent data to obtain estimates representative of the eligible primary care sample (eTable 2 in the Supplement).^35^

Unweighted characteristics of survey respondents and nonrespondents were compared using 2-sided χ^2^ tests of independence with significance set at *P* < .05. Demographic and clinical characteristics of the primary care sample (ie, survey respondents weighted to eligible primary care sample) were described on the basis of survey and EHR data. Main analyses estimated the weighted prevalence of medical cannabis use based on patient report and EHR documentation, with 95% CIs to convey precision of estimates.^36,37^ Each outcome measure was modeled using logistic regression with robust SEs^38,39^ adjusted for patient age, sex, race, ethnicity, and insurance, education, marital, employment, and residential status. Analyses of EHR-documented medical cannabis use were also adjusted for note-days.^5^ Secondary analyses repeated models for the subsample who reported any past-year cannabis use on the survey.

Patient report measures of explicit and implicit medical cannabis use were used as reference standards to evaluate the performance of EHR-documented medical cannabis use. Specifically, the weighted sensitivity, specificity, positive predictive value (PPV), and negative predictive value (NPV) of EHR-documented medical cannabis use, along with 95% CIs, were estimated with logistic regression in comparison to patient report of explicit and implicit medical cannabis use.^40^ Analyses were conducted using Stata statistical software version 15.1 (StataCorp).^41^ Data were analyzed from November 2020 to December 2021.

## Results

A total of 1688 patients responded to the survey, a mean (SD) of 77 (26) days after their index cannabis screen (mean [SD] age, 50.7 [17.5] years, 861 female [56%], 1184 White [74%], 1514 non-Hispanic [97%], and 1059 commercially insured [65%]) (Table 1). Respondents differed from nonrespondents, with a greater proportion of respondents being women, aged 65 years and older, White, and Medicare-insured compared with nonrespondents (eTable 2 in the Supplement). The main study sample included 1688 survey respondents (63% online [1063 respondents], 37% by telephone [625 respondents]) and had a 34% response rate, consistent with current health survey research.^42,43^ Weighted results, presented hereafter, indicated that the prevalence of any past-year cannabis use reported by primary care patients on the survey was 38.8% (95% CI, 31.9%-46.1%), whereas the prevalence of past-year cannabis use documented in the EHR was 21.9% (95% CI, 18.3%-26.0%) (eTable 2 in the Supplement).

**Table 1.** Characteristics of the Primary Care Sample

Characteristic	Primary care sample (N = 1688)	
	Patients, % (SE)^a^	Patients, No.^b^
Sex		
Male	44.1 (4.1)	827
Female	55.9 (4.1)	861
Age, y		
18-25	9.1 (2.4)	246
26-35	17.2 (3.1)	479
36-44	15.0 (3.2)	222
45-64	31.0 (3.9)	423
≥65	27.7 (3.4)	318
Race		
American Indian/Alaskan Native	0.1 (<1)	13
Asian/Pacific Islander	9.1 (2.3)	88
Black/African American	4.6 (1.7)	135
Multiracial^c^	3.6 (1.5)	109
Other/unknown^c^	8.4 (2.5)	158
White	74.2 (3.7)	1184
Hispanic ethnicity	3.3 (1.0)	174
Insurance		
Medicaid/subsidized	6.0 (1.8)	189
Medicare	27.1 (3.4)	323
Commercial	64.9 (3.7)	1059
Unknown	2.0 (0.8)	124
Education^d^		
Less than high school	2.8 (1.5)	34
High school graduate or GED	9.9 (2.3)	282
Some college	38.6 (4.0)	665
4-y college degree	13.4 (2.5)	378
>4-y college degree	34.4 (4.0)	316
Missing	0.9 (0.8)	10
Marital status^d^		
Married	57.0 (4.1)	695
Widowed	3.0 (1.3)	43
Divorced/separated	9.2 (2.4)	166
Single/never married	24.1 (3.5)	505
Living with partner	5.8 (1.5)	271
Missing	0.9 (0.8)	8
Employment status^d^		
Employed		
Full-time	55.4 (4.1)	988
Part-time	12.6 (2.9)	152
School/vocational	1.7 (1.1)	47
Retired	22.0 (3.1)	298
Homemaker	3.4 (1.5)	38
Unemployed	0.8 (0.2)	58
Disabled	2.4 (1.3)	73
Other	0.8 (0.6)	28
Residence^d^		
Own	67.7 (3.8)	883
Rent	28.9 (3.7)	694
Living with friends/family	2.1 (1.1)	82
No permanent residence	0.4 (0.1)	21
Missing	0.9 (0.8)	6
Frequency of past-year cannabis use (survey)^d^		
None	61.2 (3.7)	99
Less than monthly	14.6 (2.5)	99
Monthly	5.8 (1.6)	118
Weekly	7.3 (1.4)	376
Daily or almost daily	11.1 (1.7)	996

^a^ Percentage was calculated from survey data weighted for sampling and nonresponse rates for eligible primary care sample.

^b^ Number was calculated from unweighted survey data.

^c^ Patients are provided the option to indicate other when choosing among 1 or more race categories at appointing or check-in. Patients who indicated more than 1 race are reported as multiracial.

^d^ Indicates data from survey; all other data are from electronic health record.

The prevalence of patient report of explicit medical cannabis use was 26.5%; (95% CI, 21.6%-31.3%), including 15.5% (95% CI, 10.3%-19.8%) who reported medical use only and 10.9% (95% CI, 8.4%-13.4%) who reported both medical and nonmedical use (Table 2). Another 12.3% (95% CI, 9.0%-15.6%) reported nonmedical use only. The prevalence of patient report of implicit medical use (ie, any health reason for use) was 35.1% (95% CI, 29.3%-40.3%). The most common health reasons for cannabis use included pain (28.4%), sleep (19.0%), stress (19.0%), worry or anxiety (14.6%), and depression or sadness (9.6%). The prevalence of past-year EHR-documented medical cannabis use was 4.8% (95% CI, 3.45%-6.2%) (Table 2).

**Table 2.** Prevalence of Primary Care Patient Medical Cannabis Use According to Measures From a Survey and Electronic Health Record Documentation

Medical cannabis use measure	Patients, % (95% CI)^a^
Patient survey responses	
Use of cannabis in past year was for	
Nonmedical reasons	12.3 (9.0-15.6)
Medical reasons	15.5 (101.3-19.8)
Both medical and nonmedical reasons	10.9 (8.4-13.4)
Did not use cannabis in past year	61.2 (55.3-67.2)
Patient report of explicit medical cannabis use^b^	26.5 (21.6-31.3)
Use of cannabis in past year to help manage any of the following	
Pain	28.4 (23.2-33.7)
Sleep	19.0 (15.1-22.9)
Stress	19.0 (14.8-23.2)
Worry or anxiety	14.6 (11.3-17.9)
Depression or sadness	9.6 (7.2-11.9)
Muscle spasm	8.2 (5.5-10.8)
Nausea or vomiting	6.1 (4.1-8.2)
Focus or concentration	3.6 (2.4-4.8)
Appetite	3.4 (2.6-4.2)
Other	3.2 (1.6-4.7)
Seizures	0.1 (0.1-0.2)
None	3.7 (2.0-5.4)
Patient report of implicit medical cannabis use^c^	35.1 (29.3-40.8)
EHR documented measure	
Medical cannabis use (explicit and/or implicit)^d^	4.8 (3.4-6.2)

Abbreviation: EHR, electronic health record.

^a^ Percentage was calculated from survey data weighted for sampling and nonresponse rates to estimate eligible primary care sample, and adjusted for age, sex, race, ethnicity, insurance, education, marital, employment, and residential status, as well as note-days for the EHR-documented measure.

^b^ Includes report of medical only and both medical and nonmedical reasons for cannabis use.

^c^ Includes any above reasons for use except none.

^d^ EHR-documented medical cannabis use was assessed in year before the index cannabis screen documented in the EHR.

Among the 38.8% of patients who reported past-year cannabis use on the survey, the prevalence of patient report of explicit medical use was 68.1% (95% CI, 62.8%-73.5%); 40.1% (95% CI, 33.4%-46.8%) reported medical use only, and 28.2% (95% CI, 23.0%-33.3%) reported both medical and nonmedical use (eTable 3 in the Supplement). Nonmedical use only was reported by 31.8% (95% CI, 26.5%-37.0%). Among those who reported past year use, the prevalence of patient report of implicit medical use was 89.6% (95% CI, 85.6%-93.5%). Finally, 7.7% (95% CI, 6.2%-9.2%) of patients who reported past-year cannabis use on the survey had EHR-documented medical use (eTable 3 in the Supplement).

When compared with patient report of explicit medical cannabis use as the reference standard, EHR-documented medical cannabis use had a sensitivity of 10.0% (95% CI, 4.4%-15.6%), specificity of 97.1% (95% CI, 94.4%-99.8%), PPV of 55.4% (95% CI, 28.3%-82.6%), and NPV of 75.0% (95% CI, 68.9%-81.1%). Performance characteristics were similar when EHR-documented medical use was compared with patient report of implicit medical cannabis use (Table 3).

**Table 3.** Performance of EHR-Documented Medical Cannabis Use When Compared With Patient Report of Medical Use Among Primary Care Patients

Patient report^b^	EHR-documented medical cannabis use, % (95% CI)^a^			
	Sensitivity	Specificity	PPV	NPV
Explicit medical use	10.0 (4.4-15.6)	97.1 (94.4-99.8)	55.4 (28.3-82.6)	75.0 (68.9-81.1)
Implicit medical use	8.4 (4.1-12.7)	97.2 (94.1-1.00)	61.9 (33.4-90.4)	66.3 (59.3-73.3)

Abbreviations: EHR, electronic health record; NPV, negative predictive value; PPV, positive predictive value.

^a^ Percentage was calculated from survey data weighted for sampling and nonresponse rates for eligible primary care sample.

^b^ Cannabis use was determined by patient report on a survey.

## Discussion

Patients using cannabis for medical reasons may benefit from information on risks of use and evidence-based treatment alternatives. Yet, little is known about the prevalence of medical cannabis use among primary care populations or how often medical records reflect patient medical cannabis use. This survey study, conducted in a state with legal nonmedical cannabis use, found that medical cannabis use was common among primary care patients: 26.5% reported explicit medical cannabis use and 35.1% reported use of cannabis for health reasons—predominantly to manage pain, sleep, stress, anxiety, and depression. In contrast, the prevalence of EHR-documented medical cannabis use was 4.8% among all primary care patients. EHR-documented medical cannabis use had a low sensitivity for medical cannabis use when compared with patient report, identifying 10.0% or less of patients who reported explicit or implicit medical cannabis use.

To our knowledge, this is the first study to estimate the prevalence of medical cannabis use among primary care patients according to confidential patient report. Previous studies have estimated the prevalence of medical cannabis use in patients in specialty care settings,^44,45,46,47^ with specific health conditions,^47,48,49,50,51^ or recruited into research^52,53^ (range 2%-30%). This study’s survey used purposive population-based sampling, which allowed for prevalence estimates representative of primary care patients in a large health system and resulted in estimates of medical cannabis use higher than other recent surveys,^22,23,54,55,56^ yet comparable to estimates of cannabis use in states with legal nonmedical use.^57^

Asking patients about their health reasons for cannabis use may be more informative than asking explicitly about medical use. Although 26.5% of primary care patients reported medical cannabis use, 25% more patients reported a health reason for use (35.1%). These results suggest that asking patients about their use of cannabis for managing health concerns, such as pain, mood, and sleep, may identify more medical cannabis use than only asking explicitly about medical use.

This study demonstrated that most medical cannabis use is not documented in the medical record—EHR documentation missed 90% of patient self-reported cannabis use for health reasons (10.0% sensitivity equates to 90.0% of patient-reported medical use not documented). Although future exploration is needed, lack of EHR documentation may reflect absence of health system support for or prioritization of documentation, clinician priorities, and/or medical training.^58,59^ Lack of documentation may also reflect clinician reluctance to explore medical cannabis use with patients.^60,61,62^ In the study by Matson et al,^5^ patients with EHR documentation of medical use had a higher prevalence of health conditions with potential risks from cannabis use compared with patients with no or other past-year use, suggesting that patient comorbidity may also be associated with documentation.

Documentation of health reasons for cannabis use may help clinicians identify contraindications, drug interactions, and patient-initiated substitution of prescribed medications for cannabis.^15,63,64^ Moreover, documentation can support patient-centered discussions, desired by patients,^65^ about the limited benefits of cannabis use for some health conditions, insufficient evidence for cannabis use for other conditions (eg, depression and anxiety), the potential for cannabis to exacerbate or cause symptoms, and the availability of safer or more effective treatment options.^58,59,65,66,67^ Combined with cannabis screening, routinely asking about health reasons for use could improve recognition and documentation of medical cannabis use and the management of health conditions for which cannabis is being used.

### Limitations

This study has important limitations. First, 34% of invited patients completed the survey. Although this rate is consistent with nationally declining response rates, higher than industry averages for telephone (18%) and online (29%) surveys, and within the range of state-level response rates for US Centers for Disease Control and Prevention annual health-risk survey (25%-60%), it is lower than desired.^68,69^ Second, a small number of respondents represented a large number of primary care patients who did not use cannabis according to their index screen (eTable 4 in the Supplement). However, characteristics of the weighted sample reflect those of the eligible primary care sample and KPWA patients overall,^5^ suggesting weights adequately compensate for sampling and survey nonresponse. Third, this study took place within a health system that routinely screens for the frequency of any past-year cannabis use. Although screening does not ask about medical use, it could have led to discussions of patient health reasons for cannabis use.^70^ This study could not address how often such discussions occurred but were not documented. Fourth, although time between the index screen and survey was brief, changes in cannabis use could have occurred between measures. Moreover, the survey used an inclusive definition of cannabis/cannabinoid use, which was likely associated with 25% of respondents reporting past-year use on the survey despite EHR-documentation of no past-year cannabis use. Both limitations could have influenced comparisons, with patients reporting greater use on the survey than documented in the clinical setting. Additionally, although respondents reflected the demographics of primary care patients in a single health system in 1 state, findings may not generalize to other primary care populations and settings, particularly in states where cannabis use is not legal.

## Conclusions

Among primary care patients in a large integrated health system in Washington State, 35.1% of patients reported using cannabis for health reasons—predominantly pain, sleep, stress, anxiety, and depression—while 26.5% reported medical cannabis use in the past year. Only 10.0% of patients who reported medical cannabis use on the survey had medical cannabis use documented in their EHR. Asking patients about use of cannabis to manage health conditions alongside routine cannabis screening may improve recognition and documentation of medical cannabis use and the management of health conditions for which cannabis is being used.

## Notes

Supplement. **eAppendix. **Patient Survey **eTable 1.** Proportion of the Eligible Primary Care Sample Who Were Randomly Sampled and Received the Survey Within Each of 10 Strata Based on EHR-Documentation of the Frequency of Past-Year Cannabis Use and Racial and Ethnic Minoritized Group Status **eTable 2.** Comparison of Patient Characteristics for Each Sample Using Data Available From the EHR **eTable 3.** Patient Report and EHR-Documentation of Medical Cannabis Use Among Primary Care Patients Who Reported Past Year Cannabis Use on the Survey **eTable 4.** Unweighted Crosstabulation of EHR-Documented Compared With Patient Survey-Report of the Frequency of Past-Year Cannabis Use
