# Error in Role of the Funder/Sponsor

**DOI:** 10.1001/jamanetworkopen.2022.19924

**Published:** 2022-06-15

In the Original Investigation titled “Comparison of Medical Cannabis Use Reported on a Confidential Survey vs Documented in the Electronic Health Record Among Primary Care Patients,”^1^ published May 23, 2022, there was an error in the Role of the Funder/Sponsor. This section should have stated that a National Institute on Drug Abuse–appointed Data and Safety Monitoring Board (DSMB) reviewed the design and conduct of the study. This article has been corrected.^1^
