# Supplementary material for: Comparison of Medical Cannabis Use Reported on a Confidential Survey vs Documented in the Electronic Health Record Among Primary Care Patients
Source: JAMA Netw Open. 2022 May 23;5(5):e2211677. doi: 10.1001/jamanetworkopen.2022.11677 (PMC9127557; doi:10.1001/jamanetworkopen.2022.11677)
Supplement: Supplement. — eAppendix. Patient Survey eTable 1. Proportion of the Eligible Primary Care Sample Who Were Randomly Sampled and Received the Survey Within Each of 10 Strata Based on EHR-Documentation of the Frequency of Past-Year Cannabis Use and Racial and Ethnic Minoritized Group Status eTable 2. Comparison of Patient Characteristics for Each Sample Using Data Available From the EHR eTable 3. Patient Report and EHR-Documentation of Medical Cannabis Use Among Primary Care Patients Who Reported Past Year Cannabis Use on the Survey eTable 4. Unweighted Crosstabulation of EHR-Documented Compared With Patient Survey-Report of the Frequency of Past-Year Cannabis Use [file jamanetwopen-e2211677-s001.pdf]

Supplemental Online Content

Lapham GT, Matson TE, Carrell DS, et al. Comparison of medical cannabis use reported on a confidential survey vs documented in the electronic health record among primary care patients. *JAMA Netw Open*. 2022;5(5):e2211677. doi:10.1001/jamanetworkopen.2022.11677

eAppendix. Patient Survey
eTable 1. Proportion of the Eligible Primary Care Sample Who Were Randomly Sampled and Received the Survey Within Each of 10 Strata Based on EHR-Documentation of the Frequency of Past-Year Cannabis Use and Racial and Ethnic Minoritized Group Status
eTable 2. Comparison of Patient Characteristics for Each Sample Using Data Available From the EHR
eTable 3. Patient Report and EHR-Documentation of Medical Cannabis Use Among Primary Care Patients Who Reported Past Year Cannabis Use on the Survey
eTable 4. Unweighted Crosstabulation of EHR-Documented Compared With Patient Survey-Report of the Frequency of Past-Year Cannabis Use

This supplemental material has been provided by the authors to give readers additional information about their work.

© 2022 Lapham GT et al. *JAMA Network Open.*

eAppendix. Patient Survey

The MAP Study

Interviewer-Administered version of the text: GREEN FONT
Web-based version of the text: BLACK FONT

[Welcome page text]:
Welcome to the MAP Study!
Please continue reading to learn more about the study

Before we start the survey, we want to give you some information, so you are informed about your rights as a research participant. This information is on the next several pages. We will also ask you a few questions to determine if you are a good fit for the survey.

[GO TO WEB-BASED PATIENT INFORMATION FORM]

Consent
I agree to be part of this study.

[OR]

No, I don't want to be part of this study

[Patient does not consent to participate:]
Thank you for considering our research study. You may click the "back" button if you would like to continue with the survey or click the "submit" button if you are done. **[SUBMIT] [END]**

[Patient consents to participate]
Thank you for your interest in this survey! Please read ***[PHONE: listen to]*** *each question and choose the best answer. The first three questions are to find out if the study is a good fit for you. After you answer them, you are free to skip questions you don't want to answer and can end the survey at any time. [WRITTEN VERSION ONLY]: The survey will take about 15-25 minutes and, after you complete it, we will send you $20 as a thank you.*

© 2022 Lapham GT et al. *JAMA Network Open.*

Introduction:
[PHONE: I'd like to share the definition of marijuana/cannabis for this study. ***Whenever we use the term 'marijuana/cannabis' we are referring to marijuana, cannabis concentrates, edibles, lotions, ointments and tinctures made with cannabis, as well as CBD-only products.]***

Important definition of marijuana/cannabis for this survey: When we use the term 'marijuana/cannabis' we are referring to marijuana, cannabis concentrates, edibles, lotions, ointments and tinctures made with cannabis, as well as CBD-only products.

SECTION 0: Study Suitability

First, we'd like to ask some questions about marijuana/cannabis use in the past year……

Q#	Question Text	Response options	SKIP INFO	Domain	Notes
0.0	How often in the past year have you used marijuana/cannabis?	0. Never<br>1. Less than monthly<br>2. Monthly<br>3. Weekly<br>4. Daily or almost daily	REFUSAL: Participant not eligible for survey	(2) Candidate cannabis exposure measure	Required response
0.1	When did you most recently use marijuana/cannabis?	1. Never<br>2. More than a year ago	IF response Q0.0=0 and Q0.1= 1 or 2, SKIP to Q5.0 (NEVER used in past year) OTHERWISE: if response Q0.0>0 OR Q0.1≥3, Go to Q1.0		Required response<br><br>***NOTE:*** *If participant has not used in past year [**Q0.0=0 and Q0.1= 1 or 2**] they are still participants and move to section **Q5.0***

© 2022 Lapham GT et al. *JAMA Network Open.*

		3. 9 – 12 months ago<br>4. 5 – 8 months ago<br>5. 1 - 4 months ago<br>6. Past 30 days<br>7. This week<br>8. Today	**REFUSAL:** Participant not eligible for survey<br>**Otherwise: Go to Q1.0**		*to complete last section.*
0.2	When you used marijuana/cannabis during the past year, was it:	1. For medical reasons<br>2. For non-medical reasons<br>3. Both medical and non-medical reasons	**REFUSAL:** Participant not eligible for survey	(1) Medical use	**Required response**
	Error message for non-response to any of the 3 Qs above:	**Web:** An answer to this question is necessary to determine if this study is right for you. You are free to not answer or to discontinue at any time, but the first few questions are required in order to start the survey.	**Phone (Interviewer will read:** An answer to this question is necessary to determine if this study is right for you. You are free to not answer or to discontinue at any time, but the first few questions are required in order to start the survey.<br>**If respondent still declines to answer:** We will not be able to complete the survey. Thank you for thinking about being part of the study. {END CONTACT]		

© 2022 Lapham GT et al. *JAMA Network Open.*

[Patient answers the first 3 questions]

Thank you! Based on your answers, the study is a good fit for you. We'd now like to ask you questions from the survey.

SECTION 1: [Participants are past-year users, where Q0.0>0 or Q0.1≥3]

These next questions also ask about marijuana/cannabis use in the past year

1.0	During the past year, on a typical day that you used marijuana/cannabis, how many times per day did you use it?	☐ 0<br>☐ 1-2<br>☐ 3-4<br>☐ 5-9<br>☐ 10 or more			
1.1	During the past year, have you used marijuana/cannabis to help you manage any of the following:<br><br>*Check all that apply.*	[yes/no] Pain	**If yes to Pain, add Q1.1.1 if yes**	(1) Medical use	Adapted from questions sent by Rosalie Pacula
		[yes/no] Muscle spasm<br>[yes/no] Seizures<br>[yes/no] Nausea or vomiting<br>[yes/no] Sleep<br>[yes/no] Stress<br>[yes/no] Appetite<br>[yes/no] Worry or anxiety<br>[yes/no] Depression or sadness<br>[yes/no] Focus or concentration<br>☐ Other symptoms (please specify):________________<br>☐	**Skip to Q1.2**		
		☐ None of the above	**Skip to Q1.2**		

© 2022 Lapham GT et al. *JAMA Network Open.*

1.1.1	During the past-year, what type of pain did you have?	Please describe the type of pain: ______________________	**Applies if Q1.1 is checked yes for Pain**	(1) Medical use	
1.2	During the past year, did you discuss your marijuana/cannabis use with a doctor or other health care provider?	1. Yes<br>0. No		(1) Medical use	Suggested by panel
1.3	Was any of your marijuana/cannabis use in the past year based on a recommendation by any of the following?<br><br>*Check all that apply.*	☐ *Kaiser Permanente* provider (for example, doctor, physician assistant, nurse practitioner, nurse, social worker, counselor, medical assistant)<br>☐ *Non-Kaiser Permanente* provider (for example., doctor, physician assistant, nurse practitioner, nurse, social worker, counselor, medical assistant)<br>☐ In-store medical marijuana consultant<br>☐ Marijuana store staff<br>☐ Naturopath (ND)<br>☐ Family member<br>☐ Friend<br>☐ Other__[require text response]_____<br>☐ No	**Applies if Q1.2 is YES**	(1) Medical use	Separated KP vs non-KP provider to assess seeking of authorization outside of KP.<br><br>Adapted from NSDUH

© 2022 Lapham GT et al. *JAMA Network Open.*

SECTION 2 **INCLUDES: Those who used in past 30 days (reported past-year use [Q0.0scores>0)] OR used in the last 30 days [Q0.1scores=3--8])**

Thanks for telling us a little bit about your marijuana/cannabis use in the past year. Next, we'd like to ask about your marijuana/cannabis use in the past 30 days. Some questions will be like ones we just asked, but about the past 30 days.

2.0	**[Q0.1=scores 6-8]:** When you used marijuana/cannabis during the past 30 days, was it:	1. For medical reasons<br>2. For non-medical reasons<br>3. Both medical and non-medical reasons	**If response '2. For non-medical reasons', SKIP to Q2.2**	(1) Medical use	**Required response**
	**[Q0.1=scores 3-5]:** When you used marijuana/cannabis during the past 30 days, was it:	1. For medical reasons<br>2. For non-medical reasons<br>3. Both medical and non-medical reasons<br>4. I did not use marijuana/cannabis in past 30 days	**If response '2. For non-medical reasons', SKIP to Q2.2**<br><br>**If response=4 (did not use in past 30 days), SKIP to Q4.0**		
2.1	During the past 30 days, how many days did you use marijuana/cannabis for medical reasons?	_____________ days<br>**[valid range: 0-30]**		(1) Medical use	Adapted to past 30 days;
2.8	During the past 30 days, how did you use marijuana/cannabis?<br>*Please select all that apply. Did you….*	☐ Smoke it (for example, in a joint, bong, blunt, spliff or pipe)<br>☐ Vaporize it (for example, hash oil in an e-cigarette-like vaporizer, vape pen or another vaporizing device)<br>☐ Dab it (for example, using waxes or concentrates in a dab rig or other dabbing device)<br>☐ Eat it (for example, in brownies, cakes, cookies, or candy)	**If ≥2 items checked, add Q2.8.1**<br>**Otherwise skip to Section 3.**	(2) Candidate cannabis exposure measure	From BRFSS 2018

© 2022 Lapham GT et al. *JAMA Network Open.*

		☐ Drink it (for example, in a tincture, tea, cola, or alcohol)<br>☐ Apply it to skin (for example, lotion, ointment, patch, or salve)<br>☐ Use it some other way (please list):_text box answer______[autofill: “the other way(s) you mentioned’ in Q2.8.1 and Section 3]			
2.8.1	During the <u>past 30 days</u>, how did you use marijuana/cannabis <u>most often</u>?<br><br>Did you….	<u>AUTOFILL reasons checked in Q2.8:</u><br>☐ …<br>☐ …<br>☐ …<br>☐ ….<br><br>[LOGIC RULE: Allow <u>one</u> checked reason]	**Applies if ≥2 items checked in Q2.8.**	(2) Candidate cannabis exposure measure	Adapted from BRFSS
2.2	During the <u>past 30 days</u>, have you used marijuana/cannabis to help you manage any of the following:<br><br>*Check all that apply.*	[yes/no] Pain<br>[yes/no] Muscle spasm<br>[yes/no] Seizures<br>[yes/no] Nausea or vomiting<br>[yes/no] Sleep<br>[yes/no] Stress<br>[yes/no] Appetite<br>[yes/no] Worry or anxiety<br>[yes/no] Depression or sadness<br>[yes/no] Focus or concentration<br>☐ Other symptoms (please specify):_text box answer [autofill: “the other symptom(s) you mentioned’ in Q2.2.1] ________________	**Add Q2.2.1 if ≥ 2 symptoms checked**<br><br>**SKIP to Q2.2.2a if just 1 symptom checked**<br><br>**SKIP to Q2.3 if no symptoms checked**	(1) Medical use	

© 2022 Lapham GT et al. *JAMA Network Open.*

		☐ None of the above	Skip to Q2.3		
2.2.1	Please check [PHONE: tell me] the reason you use marijuana/cannabis most often during the past 30 days.	AUTOFILL reasons checked in Q2.2:<br>☐ …<br>☐ …<br>☐ …<br>☐ ….<br><br>[LOGIC RULE: Allow one checked reason]	Q2.2.1 applies if ≥ 2 reasons checked in Q2.2	(1) Medical use	Note: Q2.2.1 - Q2.2.2b are pilot questions
2.2.2a	During the past 30 days, how have you used marijuana/cannabis for **[list reason from Q2.2.1]**?<br><br>*Please select all that apply.*	☐ Smoke it (for example, in a joint, bong, blunt, spliff or pipe)<br>☐ Vaporize it (for example, hash oil in an e-cigarette-like vaporizer, vape pen or another vaporizing device)<br>☐ Dab it (for example, using waxes or concentrates in a dab rig or other dabbing device)<br>☐ Eat it (for example, in brownies, cakes, cookies, or candy)<br>☐ Drink it (for example, in a tincture, tea, cola, or alcohol)<br>☐ Apply it to skin (for example, lotion, ointment, patch, or salve)<br>☐ Use it some other way (please list):______		(1) Medical use	

© 2022 Lapham GT et al. *JAMA Network Open.*

2.2.2b	During the past 30 days, how helpful has marijuana/cannabis been for **[list reason from Q2.2.1]**?	☐ Extremely helpful<br>☐ Very helpful<br>☐ Somewhat helpful<br>☐ Slightly helpful<br>☐ Not at all helpful		(1) Medical use	
2.3	Now we would like to ask if you have used marijuana/cannabis to replace prescription medications.<br><br>During the past 30 days, did you use marijuana/cannabis to *replace*, *reduce* or *stop* use of prescribed medications?	1. Yes<br><br>0. No	**If yes, add Q2.3.1 if yes**<br><br>**If no, skip to Q2.5**	(1) Medical use	Adapted from Pacula
2.3.1	For what reasons did you use marijuana/cannabis to *replace*, *reduce*, or *stop* use of prescribed medications in the past 30 days?<br>*Check all that apply.*	[yes/no] Pain<br>[yes/no] Muscle spasm<br>[yes/no] Seizures<br>[yes/no] Nausea or vomiting<br>[yes/no] Sleep<br>[yes/no] Stress<br>[yes/no] Appetite<br>[yes/no] Irritability or grumpiness<br>[yes/no] Worry or anxiety<br>[yes/no] Depression or sadness<br>[yes/no] Focus or concentration<br>☐ Other symptoms (please specify):_______________<br>☐ None of the above	**Applies if Q2.3 is YES for substitute use**	(1) Medical use	Please provide all responses (not to be autofilled from Q2.2)
2.5	Now we would like to ask you about use of marijuana/cannabis to replace specific prescription medications.	1. Yes<br>0. No		(1) Medical use	

© 2022 Lapham GT et al. *JAMA Network Open.*

	During the past 30 days, did you use marijuana/cannabis to *replace*, *reduce* or *stop* use of opioids (such as oxycodone, codeine, Vicodin, OxyContin, methadone)?				
2.6	During the past-30 days, did you use marijuana/cannabis to *replace, reduce* or *stop* use of benzodiazepines (such as Valium, Ativan, Xanax, clonazepam)?	1. Yes<br>0. No		(1) Medical use	
2.6.5	During the past-30 days, did you use marijuana/cannabis to *replace, reduce* or *stop* use of antidepressants (such as Zoloft, Prozac, Celexa, Paxil, Lexapro, Wellbutrin, Effexor, sertraline, fluoxetine)?	1. Yes<br>0. No		(1) Medical use	

© 2022 Lapham GT et al. *JAMA Network Open.*

2.7	Was any of your marijuana/cannabis use in the past 30 days based on a recommendation by any of the following?<br><br>*Check all that apply*	☐ Kaiser Permanente provider (for example., doctor, physician assistant, nurse practitioner, nurse, social worker, counselor, medical assistant)<br>☐ Non-Kaiser Permanente provider (for example, doctor, physician assistant, nurse practitioner, nurse, social worker, counselor, medical assistant)<br>☐ In-store medical marijuana consultant<br>☐ Marijuana store staff<br>☐ Naturopath (ND)<br>☐ Family member<br>☐ Friend<br>☐ Other__[require text response]__<br>☐ No.		(1) Medical use	

© 2022 Lapham GT et al. *JAMA Network Open.*

NOTE for EXPERT PANEL: **The products endorsed in Q2.8 will be auto-filled below so that only products endorsed by the participant are asked in this section.**

Also NOTE: **It is unclear from the literature how best to ask 'how much' for marijuana/cannabis products, aside from smoking. We recognize text boxes are problematic in these instances. This is an attempt at pilot data. However,** please **provide suggestions if you know of better options for asking these questions.**

These next questions ask about your typical marijuana/cannabis use. You said that in the past 30 days, you used marijuana/cannabis in the following way(s):

AUTOFILL items checked in Q2.8

- ☐ …
- ☐ …
- ☐ …
- ☐ ….

**[AUTOFILLED for questions if** 'Smoke it' **endorsed in Q2.8]**					
3.1.a	How many days per week do you typically **smoke it**?	☐ Less than 1<br>☐ 1<br>☐ 2<br>☐ 3<br>☐ 4<br>☐ 5<br>☐ 6<br>☐ 7	**Applies if Q2.8 checked for 'Smoke it'**	(2) Candidate cannabis exposure measure	
3.2.a	On a typical day that you **smoke** marijuana/cannabis, how many times per day do you **smoke it**?	☐ Less than 1<br>☐ 1<br>☐ 2<br>☐ 3-4<br>☐ 5-9<br>☐ 10-14<br>☐ 15-19<br>☐ 20 or more		(2) Candidate cannabis exposure measure	

3.3.a	[Interviewer to say participant prior to reading question: "*For this next question, we would like for you to refer the picture included in the letter*"<br><br>On a <u>typical day</u> that you **smoke** marijuana/cannabis, <u>how much</u> do you use?<br><br>*For example, how many grams [e.g., 1/8th, 1/3rd, 1.5] of flower do you smoke in a typical day?*	____[Please provide a best guess]________ grams in a typical day<br><br>[valid: 0.00-20.00]	*See photo for grams*	(2) Candidate cannabis exposure measure	
3.4.a	What is the <u>typical THC and CBD</u> content when you **smoke** marijuana/cannabis?	☐ Higher THC and lower CBD<br>☐ Higher CBD and lower THC<br>☐ About the same THC and CBD<br>☐ Don't know		(2) Candidate cannabis exposure measure	
**[AUTOFILLED for questions if '**Vaporize it**' endorsed in Q2.8]**					
3.1.b	How many <u>days per week</u> do you typically **vaporize it**?	☐ Less than 1<br>☐ 1<br>☐ 2<br>☐ 3<br>☐ 4<br>☐ 5<br>☐ 6<br>☐ 7	**Applies if Q2.8 checked for 'Vaporize it"**	(2) Candidate cannabis exposure measure	
3.2.b	On a <u>typical day</u> that you **vaporize** marijuana/cannabis, <u>how many times per day</u> do you **vaporize it**?	☐ Less than 1<br>☐ 1<br>☐ 2<br>☐ 3-4<br>☐ 5-9<br>☐ 10-14<br>☐ 15-19<br>☐ 20 or more		(2) Candidate cannabis exposure measure	

3.3.b	On a <u>typical day</u> that you **vaporize** marijuana/cannabis, describe <u>how much</u> you **vaporize**:<br><br>*For example, how many puffs or how much of a ½ gram cartridge do you vaporize in a typical day?*	First tell us in the number of puffs you vaporize in typical day]:_______________<br>**[valid: 0.00-999.00]**<br>☐ Don't know<br><br>[Now tell us in the number of ½ gram cartridges you vaporize in typical day]:______________<br>**[valid: 0.00-99.00]**<br>☐ Don't know<br>*(This is pilot data only)*		(2) Candidate cannabis exposure measure	
3.4.b	What is the <u>typical THC and CBD</u> content when you **vaporize** marijuana/cannabis?	☐ Higher THC and lower CBD<br>☐ Higher CBD and lower THC<br>☐ About same THC and CBD<br>☐ CBD only<br>☐ Don't know		(2) Candidate cannabis exposure measure	
**[AUTOFILLED for questions if '**Dab it**' endorsed in Q2.8]**					
3.1.c	How many <u>days per week</u> do you typically **dab it**?	☐ Less than 1<br>☐ 1<br>☐ 2<br>☐ 3<br>☐ 4<br>☐ 5<br>☐ 6<br>☐ 7	**Applies if Q2.8 checked for 'Dab it'**	(2) Candidate cannabis exposure measure	
3.2.c	On a <u>typical day</u> that you **dab** marijuana/cannabis, <u>how many times per day</u> do you **dab it**?	☐ Less than 1<br>☐ 1<br>☐ 2<br>☐ 3-4<br>☐ 5-9		(2) Candidate cannabis exposure measure	

© 2022 Lapham GT et al. *JAMA Network Open.*

		☐ 10-14<br>☐ 15-19<br>☐ 20 or more			
3.3.c	On a <u>typical day</u> that you **dab** marijuana/cannabis, describe <u>how much</u> you **dab**:<br><br>*For example, how many small dabs [e.g, rice or pea size] do you dab in a typical day?*	[How much you dab in a typical day]:_]Please provide a best guess]_______________<br><br>☐ Don't know<br><br>*(This is pilot data only)*		(2) Candidate cannabis exposure measure	
3.4.c	What is the <u>typical THC and CBD</u> content when you **dab** marijuana/cannabis?	☐ Higher THC and lower CBD<br>☐ Lower THC and higher CBD<br>☐ About same THC and CBD<br>☐ CBD only<br>☐ Don't know		(2) Candidate cannabis exposure measure	
**[AUTOFILLED for questions if '**Eat it**' endorsed in Q2.8]**					
3.1.d	How many <u>days per week</u> do you typically **eat it**?	☐ Less than 1<br>☐ 1<br>☐ 2<br>☐ 3<br>☐ 4<br>☐ 5<br>☐ 6<br>☐ 7	**Applies if Q2.8 checked for 'Eat it'**	(2) Candidate cannabis exposure measure	
3.2.d	On a <u>typical day</u> that you **eat** marijuana/cannabis, <u>how many times per day</u> do you **eat it**?	☐ Less than 1<br>☐ 1<br>☐ 2<br>☐ 3-4<br>☐ 5-9<br>☐ 10-14<br>☐ 15-19<br>☐ 20 or more		(2) Candidate cannabis exposure measure	

© 2022 Lapham GT et al. *JAMA Network Open.*

3.3.d	On a <u>typical day</u> that you **eat** marijuana/cannabis, <u>how many 10 mg servings</u> do you **eat**?<br><br>*For example, how many 10 mg doses, servings or applications do you eat in a typical day?*	Number of 10 mg servings _[Please provide a best guess]____ in a typical day<br>**[valid range: 0.00-30.00]**<br><br>☐ Don't know		(2) Candidate cannabis exposure measure	
3.4.d	What is the <u>typical THC and CBD</u> content when you **eat** marijuana/cannabis?	☐ Higher THC and lower CBD<br>☐ Higher CBD and lower THC<br>☐ About same THC and CBD<br>☐ CBD only<br>☐ Don't know		(2) Candidate cannabis exposure measure	
**[AUTOFILLED for questions if 'Drink it' endorsed in Q2.8]**					
3.1.e	How many <u>days per week</u> do you typically **drink it**?	☐ Less than 1<br>☐ 1<br>☐ 2<br>☐ 3<br>☐ 4<br>☐ 5<br>☐ 6<br>☐ 7	**Applies if Q2.8 checked for 'Drink it'**	(2) Candidate cannabis exposure measure	
3.2.e	On a <u>typical day</u> that you **drink** marijuana/cannabis, <u>how many times per day</u> do you **drink it**?	☐ Less than 1<br>☐ 1<br>☐ 2<br>☐ 3-4<br>☐ 5-9<br>☐ 10-14<br>☐ 15-19<br>☐ 20 or more		(2) Candidate cannabis exposure measure	
3.3.e	On a <u>typical day</u> that you **drink** marijuana/cannabis, <u>how many 10 mg servings</u> do you **drink**?			(2) Candidate cannabis	

© 2022 Lapham GT et al. *JAMA Network Open.*

	For example, if you drink 100 mg in a typical day, you would answer "10". If you drink 50 mg in typical day, you would answer "5".	Number of 10 mg servings _[please provide a best guess]______ in a typical day<br>**[valid range: 0-30]**<br><br>☐ Don't know		exposure measure	
3.4.e	What is the typical THC and CBD content when you **drink** marijuana/cannabis?	☐ Higher THC and lower CBD<br>☐ Higher CBD and lower THC<br>☐ About same THC and CBD<br>☐ CBD only<br>☐ Don't know		(2) Candidate cannabis exposure measure	
**[AUTOFILLED for questions if 'Apply it' endorsed in Q2.8]**					
3.1.f	How many days per week do you typically **apply it**?	☐ Less than 1<br>☐ 1<br>☐ 2<br>☐ 3<br>☐ 4<br>☐ 5<br>☐ 6<br>☐ 7	**Applies if Q2.8 checked for 'Apply it'**	(2) Candidate cannabis exposure measure	
3.2.f	On a typical day that you **apply** marijuana/cannabis, how many times per day do you **apply it**?	☐ Less than 1<br>☐ 1<br>☐ 2<br>☐ 3-4<br>☐ 5-9<br>☐ 10-14<br>☐ 15-19<br>☐ 20 or more		(2) Candidate cannabis exposure measure	
3.3.f	On a typical day that you **apply** marijuana/cannabis, describe how much you **apply**:	[how much you apply in a typical day]:__[Please provide a best guess]_______________		(2) Candidate cannabis exposure measure	

© 2022 Lapham GT et al. *JAMA Network Open.*

	For example, how many pea-size drops of lotion or oil or how many patches do you apply to your skin in a day?	☐ Don't know<br>*(This is pilot data only)*			
3.4.f	What is the <u>typical THC and CBD</u> content when you **apply** marijuana/cannabis?	☐ Higher THC and lower CBD<br>☐ Higher CBD and lower THC<br>☐ About same THC and CBD<br>☐ CBD only<br>☐ Don't know		(2) Candidate cannabis exposure measure	
**[AUTOFILLED for questions if 'Other' endorsed in Q2.8]**					
3.1.g	How many <u>days per week</u> do you typically use marijuana/cannabis **another way**?	☐ Less than 1<br>☐ 1<br>☐ 2<br>☐ 3<br>☐ 4<br>☐ 5<br>☐ 6<br>☐ 7	**Applies if Q2.8 checked for 'Other'**	(2) Candidate cannabis exposure measure	
3.2.g	On a <u>typical day</u> that you use marijuana/cannabis **another way**, <u>how many times per day</u> do you **use it**?	☐ Less than 1<br>☐ 1<br>☐ 2<br>☐ 3-4<br>☐ 5-9<br>☐ 10-14<br>☐ 15-19<br>☐ 20 or more		(2) Candidate cannabis exposure measure	
3.3.g	On a <u>typical day</u> that you use marijuana/cannabis **another way**, describe <u>how much</u> you use.	[How much you use of other in a typical day]:__[Please provide a best guess]_______________<br>☐ Don't know<br>*(This is pilot data only)*		(2) Candidate cannabis exposure measure	

© 2022 Lapham GT et al. *JAMA Network Open.*

3.4.g	What is the typical THC and CBD content of the marijuana/cannabis you use **another way?**	☐ Higher THC and lower CBD<br>☐ Higher CBD and lower THC<br>☐ About same THC and CBD<br>☐ CBD only<br>☐ Don't know		(2) Candidate cannabis exposure measure	

[AUTOFILLED for questions if ≥ 2 items checked in Q2.8]

Now consider all the ways you use marijuana/cannabis....

3.1.h	How many days per week do you typically **use any** marijuana/cannabis?	☐ Less than 1<br>☐ 1<br>☐ 2<br>☐ 3<br>☐ 4<br>☐ 5<br>☐ 6<br>☐ 7	**Applies if ≥ 2 items checked in Q2.8**	(2) Candidate cannabis exposure measure	
3.2.h	On a typical day that you **use any** marijuana/cannabis, how many times per day do you **use it**?	☐ Less than 1<br>☐ 1<br>☐ 2<br>☐ 3-4<br>☐ 5-9<br>☐ 10-14<br>☐ 15-19<br>☐ 20 or more	**Applies if ≥ 2 items checked in Q2.8**		
3.3.h	What is the typical THC and CBD content of the marijuana/cannabis you use**?**	☐ Higher THC and lower CBD<br>☐ Higher CBD and lower THC<br>☐ About same THC and CBD<br>☐ CBD only<br>☐ Don't know		(2) Candidate cannabis exposure measure	

© 2022 Lapham GT et al. *JAMA Network Open.*

3.5	Before we go on, is there anything else you would like to tell us about your marijuana/cannabis use?<br><br>*For example, additional information about how or why you use marijuana/cannabis.*	Text box:_______________________<br>________________________________			

© 2022 Lapham GT et al. *JAMA Network Open.*

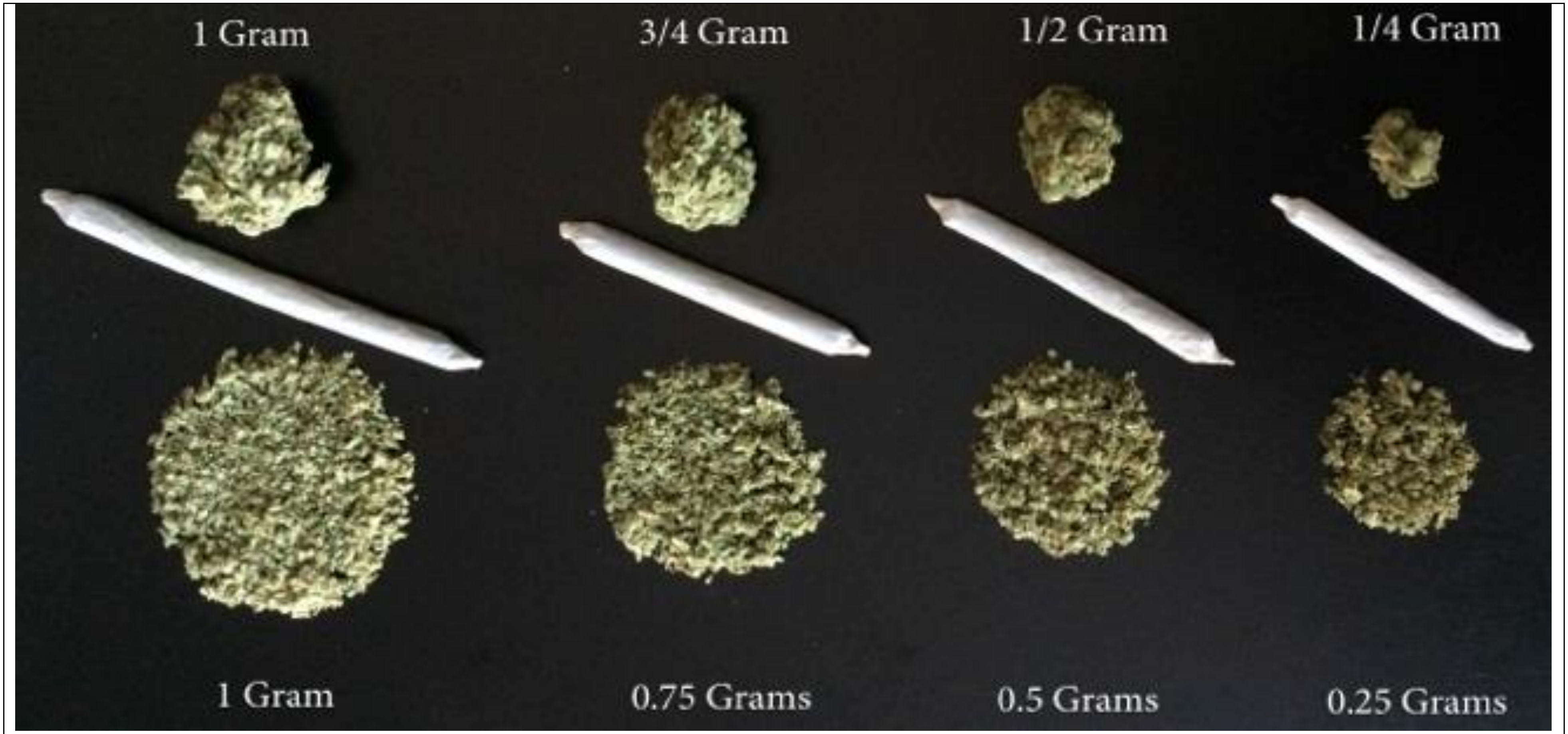

In grams: 1/8 of a gram = 0.125 grams, ¼ of a gram = 0.25 grams, ½ of a gram = 0.5 grams, ¾ of a gram = 0.75 grams.
In ounces: 1/8 of an ounce = 3.5 grams, ¼ of an ounce = 7 grams, ½ ounce = 14 grams, 1 ounce = 28 grams
NOTE: The image is true to size

© 2022 Lapham GT et al. *JAMA Network Open.*

SECTION 4

These next questions are about experiences you may have had due to marijuana/cannabis use in the past year....

4.0	In the past year, did you ever need larger amounts of marijuana/cannabis to get an effect, or did you ever find that you could no longer get high on the amount you used to use?	1. Yes<br>0. No		(3) Comparison Measure – CIDI for CUD	
4.1	Was there ever a time in the past year when you stopped, cut down, or went without using marijuana/cannabis and then experienced withdrawal symptoms?<br><br>*Withdrawal symptoms can include cravings for marijuana/cannabis, irritability, restlessness, anxiety, depression and other mood changes, sleeplessness, sweating, appetite loss, and headaches*	1. Yes<br>0. No		(3) Comparison Measure – CIDI for CUD	
4.2	Did you ever have times in the past year when you used marijuana/cannabis to keep from having withdrawal symptoms?<br><br>*Withdrawal symptoms can include cravings for marijuana/cannabis, irritability, restlessness, anxiety, depression and other mood changes, sleeplessness, sweating, appetite loss, and headaches*	1. Yes<br>0. No		(3) Comparison Measure – CIDI for CUD	
4.3	Were there times in the past year when you tried to stop or cut down on your use of marijuana/cannabis and found that you were not able to do so?	1. Yes<br>0. No		(3) Comparison Measure – CIDI for CUD	
4.4	Was there ever a time in the past year when you often had such a strong desire to use marijuana/cannabis that you couldn't stop	1. Yes<br>0. No		(3) Comparison Measure – CIDI for CUD	Also: (2) Candidate cannabis exposure measure

© 2022 Lapham GT et al. *JAMA Network Open.*

	using or found it difficult to think of anything else?				
4.5	Did you ever have times in the past year when you used marijuana/cannabis even though you planned not to or when you used a lot more than you intended?	1. Yes<br>0. No		(3) Comparison Measure – CIDI for CUD	
4.6	Were there times in the past year when you used marijuana/cannabis more frequently or for more days in a row than you intended?	1. Yes<br>0. No		(3) Comparison Measure – CIDI for CUD	
4.7	In the past year, did you ever have several days or more when you spent so much time using or getting over the effects of marijuana/cannabis use that you had little time for anything else?	1. Yes<br>0. No		(3) Comparison Measure – CIDI for CUD	
4.8	In the past year, did you ever continue to use marijuana/cannabis when you knew you had a serious physical or emotional problem that might have been caused by or made worse by using marijuana/cannabis?	1. Yes<br>0. No		(3) Comparison Measure – CIDI for CUD	
4.9	Was there ever a time in the past year when your use of marijuana/cannabis frequently interfered with your work or responsibilities at school, on a job, or at home?	1. Yes<br>0. No		(3) Comparison Measure – CIDI for CUD	
4.10	Was there ever a time in the past year when your use of marijuana/cannabis caused arguments or other serious or repeated problems with your family, friends, neighbors, or co-workers?	1. Yes<br>0. No	If no, skip to Q4.12	(3) Comparison Measure – CIDI for CUD	
4.11	Did you continue to use marijuana/cannabis even though it caused problems with these people?	1. Yes<br>0. No		(3) Comparison Measure – CIDI for CUD	

© 2022 Lapham GT et al. *JAMA Network Open.*

4.12	Were there times in the past year when you were often under the influence of marijuana/cannabis in situations where you could have gotten hurt - for example when riding a bicycle, driving, operating a machine, or anything else?	1. Yes<br>0. No		(3) Comparison Measure – CIDI for CUD	
4.13	In the past year, were there times when you experienced strong desires or cravings to use marijuana/cannabis?	1. Yes<br>0. No		(3) Comparison Measure – CIDI for CUD	
4.14	In the past year, was there ever a time when you gave up or greatly reduced important activities because of your marijuana/cannabis use - for example, sports, work, or seeing friends and family?	1. Yes<br>0. No		(3) Comparison Measure – CIDI for CUD	

© 2022 Lapham GT et al. *JAMA Network Open.*

SECTION 5

Thank you for answering the questions about marijuana/cannabis.<br>These next few questions ask specifically about your use of tobacco/nicotine products…..					
5.1	Have you ever smoked at least 100 cigarettes in your entire life?	☐ Yes	**If yes, Go to Q5.1.1**		BRFSS - cigarette use
		☐ No<br>☐ Don't know	**If No or Don't know, skip to Q5.2**		
5.1.1	Do you now smoke cigarettes …..?	☐ Every day<br>☐ Some days<br>☐ Not at all/never<br>☐ Don't know		(4) Covariates and potential confounders	BRFSS – cigs
5.2	Do you currently use chewing tobacco, snuff or snus….?	☐ Every day<br>☐ Some days<br>☐ Not at all/never<br>☐ Don't know		(4) Covariates and potential confounders	BRFSS - chewing tobacco, snuff, snus
5.3	Have you ever used an e-cigarette or other electronic vaping product for tobacco/nicotine, even just one time, in your entire life?	☐ Yes	**If yes, go to Q5.3.1**		
		☐ No<br>☐ Don't know	**If No or don't know, skip to Q5.4**		
5.3.1	Do you now use e-cigarettes or other electronic 'vaping' products for <u>tobacco/nicotine</u> …..?	☐ Every day<br>☐ Some days<br>☐ Not at all/never<br>☐ Don't know		(4) Covariates and potential confounders	BRFSS – e-cigs, vaping
5.4	Finally, a few last questions about you………	☐ Employed full-time (INCLUDES SELF-EMPLOYED)		(4) Covariates and potential confounders	

© 2022 Lapham GT et al. *JAMA Network Open.*

	What best describes your current employment? Please check the best response	☐ Employed part-time (INCLUDES SELF-EMPLOYED)<br>☐ In school or vocational training<br>☐ Retired<br>☐ Homemaker<br>☐ Unemployed, laid-off or looking for work<br>☐ Disabled or unable to work for health reasons<br>☐ OTHER [SPECIFY]________			
5.5	Are you currently…	☐ Married<br>☐ Widowed<br>☐ Divorced or Separated<br>☐ Single/ Never been married<br>☐ Living with Partner		(4) Covariates and potential confounders	
5.6	Which of the following best describes your current residence?	☐ Owned by you or someone in your immediate family<br>☐ Rented by you or someone in your immediate family<br>☐ Living with friends or extended family<br>☐ No permanent residence		(4) Covariates and potential confounders	
5.7	What is the highest grade or level of school that you completed?	☐ 8th grade or less<br>☐ Some high school, but not a graduate		(4) Covariates and potential confounders	

© 2022 Lapham GT et al. *JAMA Network Open.*

		☐ High school graduate or GED<br>☐ Some college or 2-year degree<br>☐ 4-year college degree<br>☐ More than 4-year college degree			
5.8	What is your total household income?	☐ Less than $10,000<br>☐ $10,000 to $49,999<br>☐ $50,000 to $99,999<br>☐ $100,000 to $149,999<br>☐ $150,000 or more<br>☐ Prefer not to answer		(4) Covariates and potential confounders	
5.9	"We may do other studies about marijuana/cannabis. Can we contact you about future participation?"	☐ Yes<br>☐ No<br>☐ Prefer not to answer			

[After survey completion text]
Thank you for completing the survey! We will mail your $20 thank you within the next few weeks, along with another copy of the information sheet so you have it for your records.

Where should we send it?

[WEBSITE WILL DISPLAY THE ADDRESS ON RECORD, ALLOWING THEM TO SAY IT'S THE SAME OR UPDATE, IF NEEDED.]

[ADDRESS IS AVAILABLE]: *Above is the address we have for you. Where would you like your check mailed?*

[After address is updated]:
Thank you!

We appreciate your completion of the survey. You have helped to better our understanding of marijuana/cannabis use among our patients!

© 2022 Lapham GT et al. *JAMA Network Open.*

eTable 1. Proportion of the Eligible Primary Care Sample Who Were Randomly Sampled and Received the Survey Within Each of 10 Strata Based on EHR-Documentation of the Frequency of Past-Year Cannabis Use and Racial and Ethnic Minoritized Group Status

	**EHR-documented frequency of past-year cannabis use****				
	**None**	**< monthly**	**monthly**	**Weekly**	**daily**
BIPOC					
Eligible (N)	21645	2234	662	800	1142
Sampled (N)	103	95	117	460	954
Proportion*	0.004759	0.042525	0.176737	0.575000	0.835377
	**None**	**< monthly**	**monthly**	**weekly**	**daily**
White/unknown race					
Eligible (N)	63,083	8,513	2,868	3,538	4,465
Sampled (N)	197	205	206	717	1,946
Proportion*	0.003123	0.024081	0.071827	0.202657	0.435834

*Proportion=(sampled N/eligible N), weights were created from inverse of the proportion (inverse=1/[proportion]); **EHR documented frequency of past-year cannabis use based on index cannabis screen. Racial and ethnic minoritized groups include Black, Indigenous, and other patients of color, including Hispanic/Latinx ethnicity patients.

© 2022 Lapham GT et al. *JAMA Network Open.*

**eTable 2.** Comparison of Patient Characteristics for Each Sample Using Data Available From the EHR							
			**Survey Samples**				
		**Eligible primary care patients**[a]	**Eligible survey sample**[b]	**Survey non-respondents**	**Survey respondents**	**p-value**[c]	**Primary Care Sample**[d]
		(N=108,950)	(N=5,000)	(N=3,312)	(N=1,688)		(N=1,688)
		%		%	%		%
**Sex**							
	Male	42.5	51.6	53.0	49.0	0.008	44.1
	Female	57.5	48.4	47.0	51.0		55.9
**Age**							
	18-25	8.8	16.7	17.7	14.6	<0.001	9.1
	26-35	15.6	30.1	30.9	28.4		17.2
	36-44	12.7	14.8	15.7	13.2		15.0
	45-64	35.6	25.8	26.1	25.1		31.0
	65+	27.4	12.7	9.5	18.8		27.7
**Race**							
	American Indian/Alaskan Native	0.7	1.5	1.8	0.8		0.1
	Asian/Pacific Islander	10.9	7.3	7.7	5.2	<0.001	9.1
	Black/African American	4.9	9.2	9.8	8.1		4.6
	Multiracial	2.7	7.2	8.2	6.5		3.6
	Other/Unknown	9.6	11.8	12.7	9.4		8.4
	White	71.2	63.1	59.8	70.1		74.2
**Hispanic ethnicity**		6.0	11.0	11.3	10.3	0.292	3.3
**Insurance**							
	Medicaid/Subsidized	7.5	10.5	10.2	11.2	<0.001	6.0
	Medicare	26.5	12.5	9.3	18.7		27.1
	Commercial	60.2	69.6	73.1	62.7		64.9
	Unknown	5.8	7.4	7.4	7.3		2.0
**Frequency of cannabis use (EHR-documented)**[e]							
	Never	77.8	6.0	6.1	5.9	0.487	78.1
	Less than monthly	9.9	6.0	6.1	5.9		9.6
	Monthly	3.2	6.5	6.2	7.0		3.3
	Weekly	4.0	23.5	24.2	22.3		4.0
	Daily/almost daily	5.2	58.0	57.5	59.0		5.1

[a]Adult primary care patients with a cannabis screen documented as part of routine primary care; [b]Primary care patients offered the survey, oversampled for higher frequency of past-year cannabis use and Black, Indigenous, and patients of color, including Hispanic/Latinx patients; [c]Chi-square tests of independence for differences between survey nonrespondents and respondents; [d]Based on survey data weighted for sampling and non-response to estimate the eligible primary care sample; [e]EHR-documented frequency of past-year cannabis use based on index cannabis screen

© 2022 Lapham GT et al. *JAMA Network Open.*

eTable 3. Patient Report and EHR-Documentation of Medical Cannabis Use Among Primary Care Patients Who Reported Past Year Cannabis Use on the Survey

	**Primary care patients who reported past-year cannabis use**[a] (n=1589)	
	%	(95% CI)
Patient survey responses		
Use of cannabis in past-year was for:		
Non-medical reasons	31.8	(26.5-37.0)
Medical reasons	40.1	(33.4-46.8)
Both medical and non-medical reasons	28.2	(23.0-33.3)
Did not use cannabis in past-year	--	--
**Patient report of explicit medical cannabis use**[b]	**68.1**	**(62.8-73.5)**
Use of cannabis in past-year to help manage any of the following:		
Pain	73.3	(68.3-78.3)
Sleep	48.9	(44.1-53.6)
Stress	49.1	(41.8-56.3)
Worry or anxiety	37.7	(31.8-43.6)
Depression or sadness	24.7	(20.9-28.4)
Muscle spasm	21.0	(16.2-25.9)
Nausea or vomiting	15.8	(12.1-19.5)
Focus or concentration	9.3	(6.5-12.0)
Appetite	8.7	(7.0-10.4)
Other	8.3	(4.9-11.7)
Seizures	0.5	(0.2-0.8)
None	10.4	(6.5-14.4)
**Patient report of implicit medical cannabis use**[c]	**89.6**	**(85.6-93.5)**
EHR-documented measure		
**EHR-documented medical cannabis use (explicit and/or implicit)**[d]	**7.7**	**(6.2-9.2)**

[a]Based on survey data weighted for sampling and non-response rates and adjusted for age, sex, race, ethnicity, insurance, education, marital, employment, and residential status, as well as note-days for the EHR-documented measure; [b]Includes report of 'medical only' and 'both medical and non-medical reasons' for cannabis use; [c]Includes any above reasons for use except 'none'; [d]EHR-documented medical cannabis use was assessed in year prior to the index cannabis screen documented in the EHR

© 2022 Lapham GT et al. *JAMA Network Open.*

eTable 4. Unweighted Crosstabulation of EHR-Documented Compared to Patient Survey-Report of the Frequency of Past-Year Cannabis Use

	**Patient survey-report of the frequency of past-year cannabis use** (n=1,688)					
**EHR-documented frequency of past-year use**	Never	Less than monthly	Monthly	Weekly	Daily or almost	Total
	N	N	N	N	N	N
Never	74	13	3	4	5	99
Less than monthly	4	57	17	16	5	99
Monthly	4	14	36	35	29	118
Weekly	7	12	32	161	164	376
Daily or almost	6	9	16	62	903	996
Total	95	105	104	278	1,106	1,688

EHR=electronic health record
